# Association between BMI and knee osteoarthritis in Chinese adults aged 45 years and older: evidence from the 2021 Global Burden of Disease analysis and the China Health and Retirement Longitudinal Study

**DOI:** 10.3389/fpubh.2025.1738564

**Published:** 2026-01-16

**Authors:** Haiyan Luo, Xiaoyue Zhao, Xian Lei, Jun Ma, Xue Wang, Yu Zhao, Yaogeng Chen

**Affiliations:** 1 School of Public Health, Ningxia Medical University, Yinchuan, China; 2 School of Medical Information and Engineering, Ningxia Medical University, Yinchuan, China

**Keywords:** body mass index, chronic disease control, disease burden, knee osteoarthritis, weight-management

## Abstract

**Background:**

Amidst the dual challenges of rapid population aging and rising obesity prevalence, knee osteoarthritis (KOA) constitutes a major public health burden in China. This study examined the association between body mass index (BMI) and KOA in Chinese adults, and aimed to identify clinically meaningful BMI thresholds for weight management interventions.

**Methods:**

Using data from the Global Burden of Disease 2021 Study (1990–2021), we quantified the KOA burden attributable to high BMI and assessed age-specific temporal trends. We further analyzed data from the China Health and Retirement Longitudinal Study (CHARLS), including participants aged ≥45 years after excluding those with missing BMI, KOA, or covariate information and those with BMI values outside 15–40 kg/m^2^. Restricted cubic spline modelling combined with piecewise linear regression was used to evaluate the non-linear relationship between BMI and KOA risk and to identify potential threshold effects.

**Results:**

In 2021, China recorded 1.11 million disability-adjusted life years (DALYs) attributable to high BMI among individuals with KOA, a 369.28% increase from 1990. After age standardization, the DALYs rate increased by 91.68%, with an average annual increase of 2.13% (*p* < 0.05). The crude DALYs rate in adults aged ≥45 years rose markedly from 1990 to 2021 and continued to increase with age. Among 12,769 CHARLS participants, 1,254 (9.82%) were diagnosed with KOA. The proportion of overweight and obesity were significantly higher in the KOA group than in the non-KOA group (*p* < 0.001). After adjustment for demographic and health-related covariates, KOA prevalence increased monotonically with rising BMI. Piecewise regression analyses detected a non-linear association between BMI and KOA prevalence, with a threshold at 26.89 kg/m^2^. Above this threshold, each 1-unit BMI increase corresponded to a 10% rise in KOA prevalence [odds ratio (OR) = 1.10, 95% confidence interval (CI): 1.07–1.13, *p* < 0.001].

**Conclusion:**

A BMI of 26.89 kg/m^2^ represents a significant inflection point for KOA risk among Chinese adults aged ≥45 years. Tiered weight-management strategies centered on this threshold, integrated with population-based chronic disease control initiatives, are recommended to halt the ongoing increase in KOA-related burden.

## 1 Introduction

Osteoarthritis (OA) is a chronic, degenerative joint disorder characterised by progressive cartilage degradation and low-grade synovial inflammation (1). Knee osteoarthritis (KOA) is the most prevalent phenotypic subtype of OA (2), and its global prevalence is projected to increase in prevalence by 74.9% by 2050 (3). KOA causes persistent pain and functional impairment in patients, significantly compromising patients’ quality of life and imposing a substantial socioeconomic burden (4). Despite the large number of individuals affected by KOA, current treatment methods primarily focus on alleviating symptoms, with analgesics and anti-inflammatories being the mainstay of therapy. Joint arthroplasty is typically reserved for end-stage disease (5). Such end-stage interventions are inherently incapable of reversing disease progression and therefore represent a pressing public health challenge requiring urgent attention (6).

Against the backdrop of a global ageing population, China—with its large population undergoing rapid demographic change—provides a unique perspective on quantifying the burden of KOA (7). By 2050, the proportion of older adults in China is expected to more than double, leading to a corresponding increase in age-related conditions such as KOA and putting unprecedented strain on its healthcare system (8). Concurrently, rapid economic expansion, accelerating globalisation and relentless urbanisation have caused a significant increase in the prevalence of overweight and obesity among Chinese adults (9). When indexed using body mass index (BMI), adiposity constitutes a readily modifiable risk factor. Cross-sectional analyses by Lv et al. (10) revealed a monotonic gradient between successively higher BMI strata and the prevalence of radiographic KOA. Shao et al. (11) quantified this association, reporting odds ratio of 1.91 for overweight individuals and 4.63 for obese individuals relative to normal-weight individuals. Similar findings reported by Ji et al. (12) yielded corresponding multivariable-adjusted relative risk estimates of 1.51 and 2.24, respectively. Despite the consistency of these findings, existing investigations have predominantly operationalised BMI as a categorical exposure variable, partitioning the continuous adiposity distribution according to conventional epidemiological cut-points. This analytical discretisation inherently constrains insight into potential nonlinearity and obscures fine-grained dose–response relationships. Meanwhile, although a growing body of literature has sought to contextualise the burden of KOA attributable to elevated BMI in China (7, 13, 14), quantitative elucidation of the precise functional form linking BMI to KOA risk remains limited. This evidence gap restricts the formulation of optimally targeted, metric-specific public health interventions.

Therefore, to generate actionable evidence for guiding public health strategy development and alleviating the growing burden of KOA in ageing societies, this study used Global Burden of Disease (GBD) 2021 data to describe the burden of KOA attributed to high BMI among Chinese adults. We observed that the burden of disability-adjusted life years (DALYs) increased markedly among individuals aged 45 years and older. To further elucidate the relationship between BMI and KOA in this population, we analyzed data from the China Health and Retirement Longitudinal Study (CHARLS) among adults aged 45 years and over to quantify the impact of BMI on KOA prevalence. Our findings indicate that once BMI exceeds 26.89 kg/m^2^, each additional unit increase is associated with a 10% increase in KOA prevalence. However, because this study relies on cross-sectional CHARLS data collected in 2011, the findings may not fully reflect the contemporary relationship between BMI and KOA among middle-aged and older adults in China. Accordingly, extrapolation of these results should be interpreted with caution.

## 2 Materials and methods

### 2.1 Data collection

#### 2.1.1 GBD 2021

The GBD 2021 study provides annual estimates of the incidence, prevalence and mortality of 370 diseases and injuries, as well as estimates of years of life lost (YLLs), years lived with disability (YLDs) and DALYs, for 204 countries and territories, covering the period from 1990 to 2021 (15–17). Within the GBD 2021 framework, incident and prevalent cases of OA are defined as symptomatic disease corroborated by radiographic evidence and graded as Kellgren–Lawrence 2–4. The primary data sources for KOA include population-based cross-sectional surveys conducted worldwide and U.S. state-level administrative claims databases. Cases were identified using the International Classification of Diseases, Tenth Revision (ICD-10) code M17 (knee joint) (18). It is notable that high BMI is the only modifiable risk factor for osteoarthritis explicitly modelled in the GBD dataset. Meanwhile, high BMI among adults aged ≥20 years was operationally defined as any measured value exceeding the theoretical minimum risk exposure level (TMREL) of 20–23 kg/m^2^, a range identified by the GBD consortium through comparative risk assessment (19).

Epidemiological data on the incidence, prevalence, DALYs, age-standardized incidence (ASIR), age-standardized prevalence (ASPR), age-standardized DALYs rate (ASDR) and KOA-related risk factors were obtained from the GBD Results Tool.^1^ All individual-level GBD data were fully de-identified, and the University of Washington Institutional Review Board waived the requirement for informed consent. Consequently, no additional ethical approval was required for the present analyses.

#### 2.1.2 CHARLS

CHARLS is a nationally representative longitudinal survey of Chinese adults aged ≥45. Using a multistage, probability-proportional-to-size sampling design, the study covered 150 county-level units across 28 provinces (20). Comprehensive face-to-face interviews and standardized physical examinations were conducted to collect detailed information on demographics, health status, socioeconomic position, and lifestyle behaviours. The CHARLS data are publicly available at http://charls.pku.edu.cn/.

In CHARLS, participants were classified as having KOA only if they met all three of the following criteria:

(1) An affirmative response to the question, “Have you ever been told by a doctor that you have arthritis or rheumatism?”
(2) An affirmative response to “Are you often troubled by any bodily pain?” and
(3) Designation of the knee as the primary site of pain (21).

BMI was calculated by dividing weight in kilograms divided by the square of height in metres (kg/m^2^). In CHARLS, height and weight were measured during the field survey. Values outside the range of 130–200 cm for height and 30–150 kg for weight were discarded as implausible. To safeguard analytical robustness, we restricted BMI to the 15–40 kg/m^2^ interval, thereby excluding extreme outliers while remaining consistent with prior investigations of BMI-disease relationships and reflecting the empirical distribution observed within CHARLS (22). For categorical analyses, BMI was categorised into four groups: <18.5 kg/m^2^, 18.5–23.9 kg/m^2^, 24.0–27.9 kg/m^2^ and ≥28.0 kg/m^2^.

Additional variables included age (continuous, in years), sex (male/female), educational attainment (illiterate, primary, junior high school, senior high school or above), marital status (married or cohabiting versus other, encompassing married but not cohabiting, separated, divorced, widowed or never married), smoking behaviour (current or former smokers versus never smokers), alcohol consumption (defined as consuming alcohol more than once per month versus never or infrequent drinkers [<once per month or never]) and physician-diagnosed chronic conditions (hypertension, diabetes mellitus, renal disease, dyslipidaemia and gastrointestinal or other digestive disorders, all ascertained by self-report). Sex (male/female) was self-reported by participants and no additional information on gender identity was collected. All variables were obtained through standardised household interview questionnaires. The CHARLS protocol was approved by the Peking University Institutional Review Board (IRB00001052-11015), and written informed consent was obtained from all participants.

### 2.2 Statistical analysis

#### 2.2.1 Trends in the KOA and the contribution attributable to high BMI in China

First, we employed a cross-sectional analysis to describe the current disease burden of KOA in China in 2021. We then characterised temporal trends using continuous estimates from 1990 to 2021. In an attributable risk assessment, we used relative risk estimates in conjunction with the population attributable fraction (PAF) to quantify the relationship between BMI and KOA burden (23). This approach enabled a systematic evaluation of the disease burden attributable to high BMI in China over the study period, as well as changes in this burden over time. Temporal trends were evaluated using Joinpoint regression analysis (Joinpoint Regression Program, JRP), which summarises the overall direction and magnitude of change as the average annual percentage change (AAPC).


$$\text{AAPC}=\{\exp(\frac{\sum \omega_{i}\beta_{i}}{\sum \omega_{i}})-1\}\times 100$$ (1)

Within the JRP framework, *β* represents the segment-specific regression coefficient, $\omega_{i}$ denotes the number of years encompassed by each segment, and $\beta_{i}$ indexes the corresponding segment. If the AAPC is greater than zero, it indicates an increasing trend over the study period, whereas a negative AAPC indicates a decreasing trend. The Joinpoint regression program (version 5.2.0), developed by the National Cancer Institute, was used for regression analysis.

#### 2.2.2 Analysis of the nonlinear relationship between BMI and KOA in Chinese adults aged 45 and above

This study utilised baseline survey data from 2011 initially comprising 17,705 participants. Following sequential exclusion of individuals with missing data on BMI, KOA status, age, gender, smoking status, alcohol consumption, hypertension, diabetes, kidney disease, digestive system disorders, and dyslipidaemia, alongside the removal of extreme outliers, the final analysis cohort comprised 12,769 subjects (see Figure A1 for the participant flow diagram). The baseline characteristics of CHARLS participants were summarised as the mean ± standard deviation for continuous variables and as the counts (percentage) for categorical variables. Comparisons between the KOA and non-KOA groups were conducted using the Wilcoxon rank-sum test for continuous variables and the chi-squared or Fisher’s exact test for categorical variables, as appropriate.

To evaluate the potential non-linear association between BMI and KOA risk, we used restricted cubic splines (RCS) regression to fit a smooth dose–response curve within the CHARLS cohort. The model was adjusted *a priori* for the following confounding factors: age, sex, marital status, educational attainment, smoking status, alcohol consumption, hypertension, diabetes mellitus, renal disease, dyslipidaemia and digestive disorders. We then implemented a piecewise regression model and used the likelihood-ratio test to compare the linear versus non-linear specifications, thereby identifying potential threshold effects.

Finally, we conducted sensitivity analyses by stratifying the entire sample at the derived BMI cut-off point to confirm the robustness of the observed associations. All statistical analyses and graphical presentations were performed using R (version 4.4.1). All tests were two-sided, and statistical significance was defined as *α* = 0.05, with *p* < 0.05 considered statistically significant.

## 3 Results

### 3.1 Description of the disease burden of KOA in China in 2021

In 2021, China recorded approximately 8.51 million incident cases, 109.58 million prevalent cases, and 3.55 million DALYs attributable to KOA. The corresponding age standardized rates per 100,000 people were 406.42 [95% uncertainty interval (UI): 348.70–467.23] for ASIR, 5016.52 (95% UI: 4265.22–5758.38) for ASPR, and 162.44 (95% UI: 78.35–314.13) for ASDR (see Table 1). Figure A2 showed the absolute counts and crude rates of incident cases, prevalent cases, and DALYs attributable to KOA in China in 2021, disaggregated by sex and 5-year age groups. The crude incidence rate increased sharply between the ages of 35 and 49, rose markedly after the age of 40, and peaked in the 50–54 age group. Crude prevalence, on the other hand, exhibited a monotonic upward trajectory with advancing age. Across all metrics, females consistently experienced higher burdens than males, highlighting the importance of incorporating sex-specific considerations into KOA prevention and management strategies.

**Table 1:** Number of cases, age-standardized incidence, prevalence and DALYs rate for KOA across all age groups in China in 2021.

Indicates	All ages cases (*n*, 95% UI)			Age standardized rates per 100,000 people (*n*, 95% UI)		
	Both	Male	Female	Both	Male	Female
Incidence	8,512,397 (7,279,974, 9,840,885)	3,184,666 (2,708,000, 3,707,256)	5,327,731 (4,573,927, 6,146,551)	406.42 (348.70, 467.23)	304.92 (261.11, 351.75)	508.53 (436.60, 583.43)
Prevalence	109,575,472 (92,723,351, 126,639,049)	39,280,537 (33,096,648, 45,575,847)	70,294,936 (59,713,905, 80,953,335)	5,016.52 (4,265.22, 5,758.38)	3,661.85 (3,106.11, 4,228.87)	6,302.93 (5,378.56, 7,213.70)
DALYs	3,554,153 (1,715,777, 6,842,994)	1,284,863 (614,830, 2,467,314)	2,269,291 (1,097,550, 4,397,450)	162.44 (78.35, 314.13)	119.45 (57.51, 229.15)	203.49 (98.22, 395.51)

DALYs, disability-adjusted life years; 95% UI, 95% uncertainty interval.

### 3.2 Changes in the disease burden of KOA in China from 1990 to 2021

**Figure 1:**
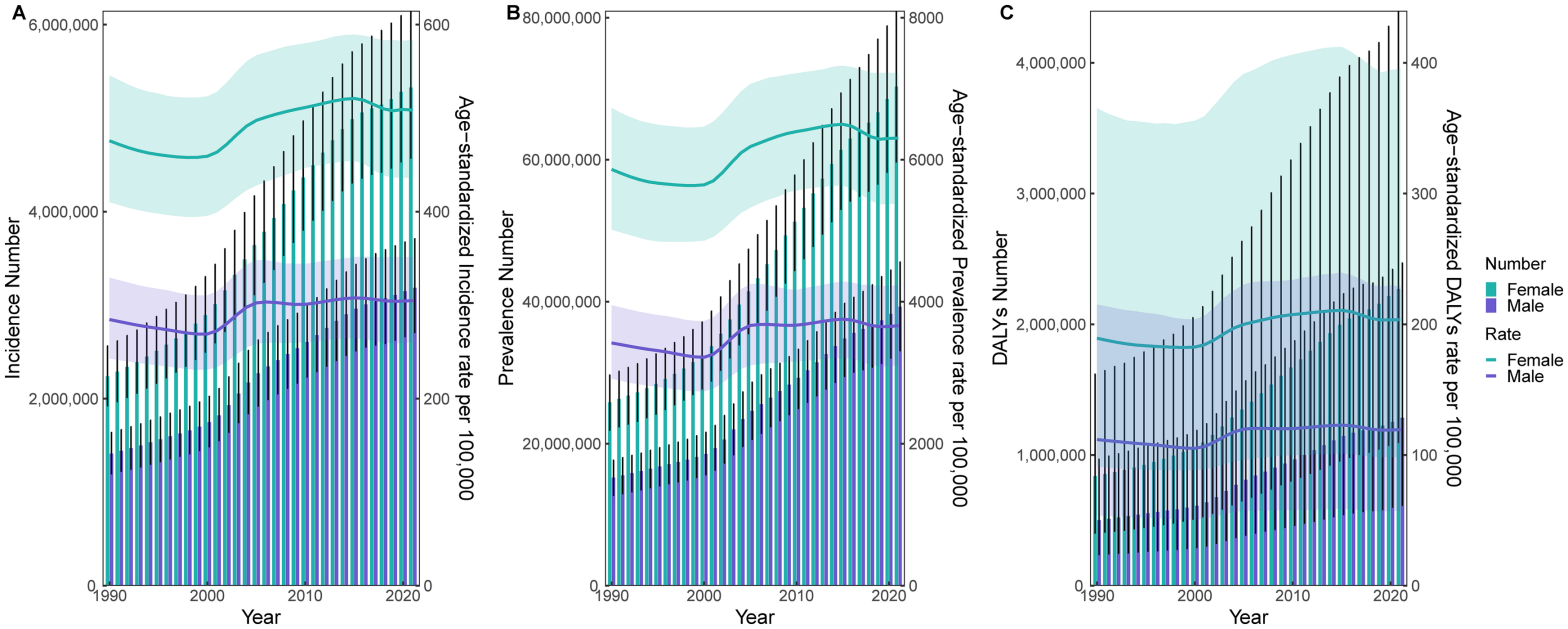
Changes in the disease burden of KOA by gender in China from 1990 to 2021. **(A)** Changes in incidence number and age-standardized rate. **(B)** Changes in prevalence number and age-standardized rate. **(C)** Changes in DALYs number and age-standardized rate.

Between 1990 and 2021, the incident cases of KOA in China increased from 3,650,857 to 8,512,397, while the prevalent cases rose from 41,044,009 to 109,575,472. DALYs attributable to KOA increased from 1,339,566 to 3,554,153 person-years. Compared with 1990, the ASIR, ASPR, and ASDR of KOA in 2021 exhibited modest upward trends despite minor fluctuations (see Table A1). For both sexes, the absolute numbers of incident cases, prevalent cases, and DALYs, as well as the corresponding ASIR, ASPR, and ASDR, increased continuously over the study period, with consistently higher values observed among females than males (see Figure 1).

### 3.3 KOA disease burden in China attributable to high BMI from 1990 to 2021

In 2021, KOA in China accounted for 1.11 million DALYs attributable to high BMI, marking a 369.28% increase since 1990. Among men, attributable DALYs rose from 80.8 thousand to 363.0 thousand (an 349.29% increase), while among women they increased from 156.1 thousand to 748.6 thousand (an increase of 379.63%). The crude DALYs rate per 100,000 people increased from 20.13 to 78.13 (a rise of 288.05%), with the rate for men rising from 13.31 to 49.85 and the rate for women rising from 27.40 to 107.77, approximately twice the rate for men.

**Figure 2:**
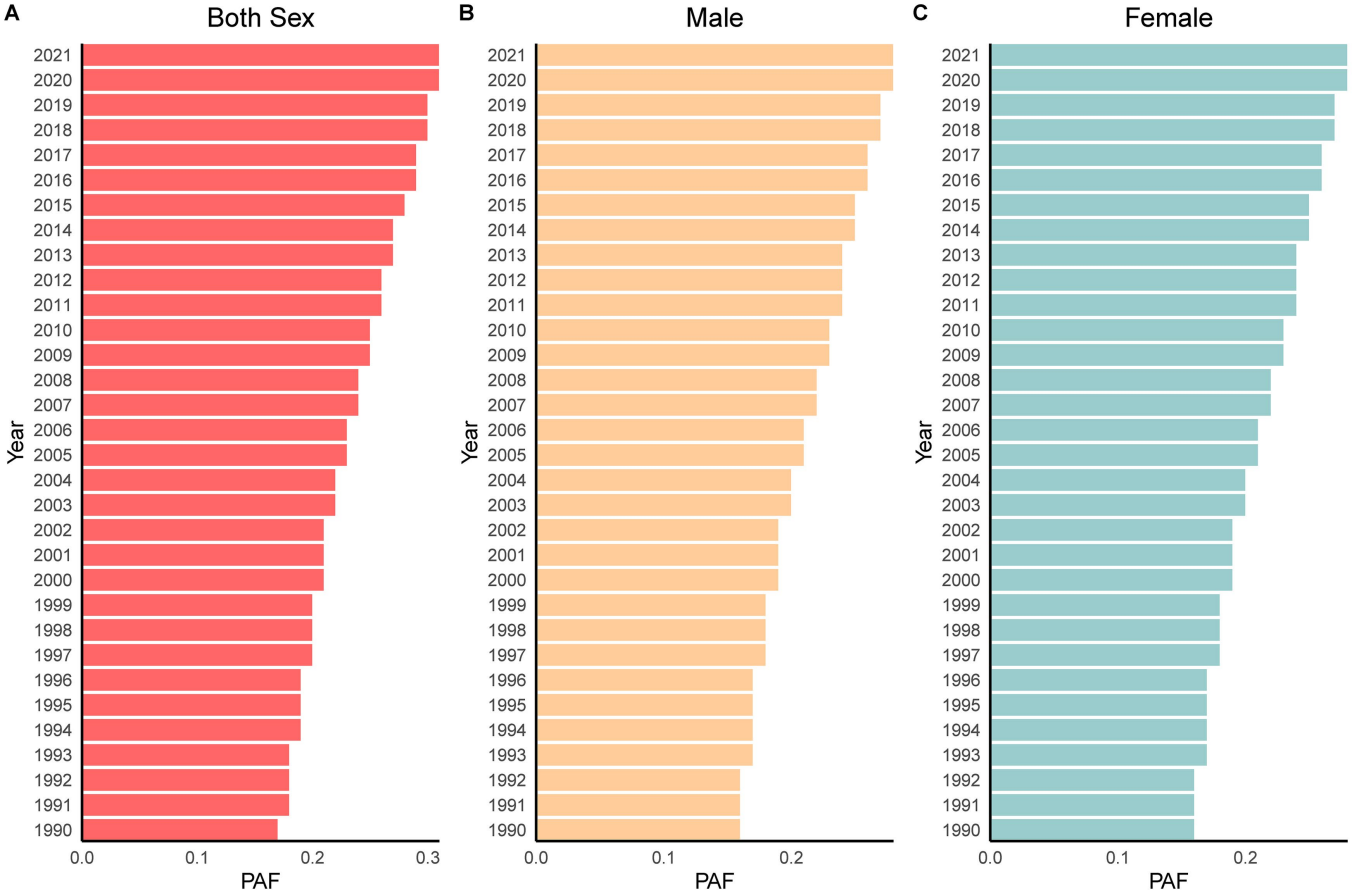
Attribution proportion of high BMI in China KOA DALYs from 1999 to 2021. **(A)** Changes in high BMI attribution among the both sex population. **(B)** Changes in high BMI attribution among the male population. **(C)** Changes in high BMI attribution among the female population.

After age standardization, the attributable DALYs rate continued to show an obvious upward trend, increasing by 91.68% overall and yielding an AAPC of 2.13% (*p* < 0.05). This increase was marginally steeper in women (AAPC = 2.12%, *p* < 0.05) than in men (AAPC = 2.10%, *p* < 0.05). In 2021, 0.31% of total KOA DALYs were attributable to high BMI, representing a 78.50% increase since 1990. Notably, women consistently bore a greater attributable burden than men. Overall, from 1990 to 2021, China experienced a sustained increase in the KOA burden attributable to elevated BMI, with the female population being affected disproportionately (see Table 2 and Figure 2).

**Table 2:** Disease burden of KOA attributable to high BMI in China, 1990–2021.

Population group	DALYs number (thousand person-years)	Crude DALYs rate (per 100,000)	Age-standardized DALYs rate (per 100,000)	PAF (%)	AAPC (%)
Total population					2.13^*^
1990	23.69	20.13	26.34	0.17	
2021	111.16	78.13	50.49	0.31	
Relative change (%)	369.28	288.05	91.68	78.50	
Male					2.10^*^
1990	8.08	13.31	17.65	0.16	
2021	36.30	49.85	33.54	0.28	
Relative change (%)	349.29	274.46	90.06	77.86	
Female					2.12^*^
1990	15.61	27.40	34.90	0.18	
2021	74.86	107.77	66.80	0.33	
Relative change (%)	379.63	293.30	91.42	78.16	

DALYs, disability-adjusted life years; PAF, population attributable fractions; AAPC, average annual percent change; ^*^p < 0.05.

**Figure 3:**
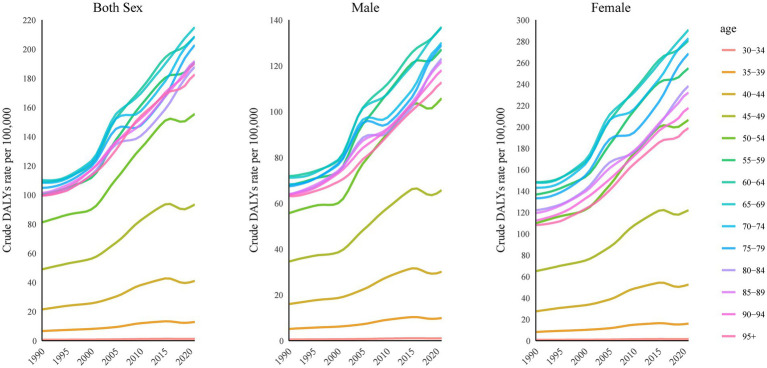
Temporal trends in the burden of KOA attributable to high BMI across age groups in China, 1990–2021.

Between 1990 and 2021, the crude DALYs rate attributed to KOA and high BMI increased unevenly across all age groups in China, with significant differences in the extent of the increase between age groups. Among adults aged 30–34, 35–39, and 40–44 years, the increase was relatively modest, with shallow slopes and limited absolute changes. In contrast, from age 45 years onward, the crude DALYs rate rose sharply, and the attributable burden intensified progressively with advancing age (see Figure 3).

### 3.4 Demographic and clinical characteristics of adults aged ≥45 years with KOA in the CHARLS

A total of 12,769 participants aged 45 years or older were included in the study, of whom 1,254 (9.82%) had KOA. The mean age of the KOA group was significantly higher than that of the non-KOA group (60.89 ± 9.05 vs. 59.29 ± 9.62 years). KOA cases were predominantly female (66.67%), whereas men accounted for only 33.33% of cases. Illiteracy was more prevalent in the KOA group (37.08%), while only 4.47% had completed senior high school or above. Participants who were unmarried, divorced, or widowed were slightly over-represented in the KOA cohort (19.94% vs. 17.14%). Overweight and obesity were more prevalent among those with KOA.

Compared with non-KOA participants, individuals with KOA had lower smoking and alcohol consumption rates (33.49% vs. 40.76 and 19.94% vs. 25.51% respectively), but higher hypertension (30.94% vs. 23.37%), diabetes mellitus (7.74% vs. 5.62%), renal disease (16.51% vs. 5.25%), gastrointestinal disorders (44.98% vs. 20.47%), and dyslipidaemia (11.56% vs. 8.75%) prevalence rates. All demographic, lifestyle, and comorbidity indicators differed significantly between the KOA and non-KOA groups (*p* < 0.05) (see Table 3).

**Table 3:** Demographic and baseline characteristics of KOA group versus non-KOA group.

Variable	All participants (*n* = 12,769)	Non-KOA (*n* = 11,515)	KOA (*n* = 1,254)	*p*-value
Age	59.44 ± 9.58	59.29 ± 9.62	60.89 ± 9.05	<0.001
Gender				<0.001
Male	6,101 (47.78%)	5,683 (49.35%)	418 (33.33%)	
Female	6,668 (52.22%)	5,832 (50.65%)	836 (66.67%)	
Education				<0.001
Illiterate	3,566 (27.93%)	3,101 (26.93%)	465 (37.08%)	
Primary school	5,217 (40.86%)	4,650 (40.38%)	567 (45.22%)	
Middle school	2,606 (20.41%)	2,440 (21.19%)	166 (13.24%)	
High school and above	1,380 (10.81%)	1,324 (11.50%)	56 (4.47%)	
Marital status				0.013
Married	10,545 (82.58%)	9,541 (82.86%)	1,004 (80.06%)	
Others	2,224 (17.42%)	1,974 (17.14%)	250 (19.94%)	
BMI				<0.001
Underweight	843 (6.6%)	748 (6.50%)	95 (7.58%)	
Normal	6,815 (53.37%)	6,173 (53.61%)	642 (51.20%)	
Overweight	3,711 (29.06%)	3,372 (29.28%)	339 (27.03%)	
Obesity	1,400 (10.96%)	1,222 (10.61%)	178 (14.19%)	
Smoke				<0.001
Yes	5,114 (40.05%)	4,694 (40.76%)	420 (33.49%)	
No	7,655 (59.95%)	6,821 (59.24%)	834 (66.51%)	
Drink				<0.001
Yes	3,188 (24.97%)	2,938 (25.51%)	250 (19.94%)	
No	9,581 (75.03%)	8,577 (74.49%)	1,004 (80.06%)	
Hypertension				<0.001
Yes	3,079 (24.11%)	2,691 (23.37%)	388 (30.94%)	
No	9,690 (75.89%)	8,824 (76.63%)	866 (69.06%)	
Diabetes mellitus				0.002
Yes	744 (5.83%)	647 (5.62%)	97 (7.74%)	
No	12,025 (94.17%)	10,868 (94.38%)	1,157 (92.26%)	
Renal diseases				<0.001
Yes	812 (6.36%)	605 (5.25%)	207 (16.51%)	
No	11,957 (93.64%)	10,910 (94.75%)	1,047 (83.49%)	
Gastrointestinal diseases				<0.001
Yes	2,921 (22.88%)	2,357 (20.47%)	564 (44.98%)	
No	9,848 (77.12%)	9,158 (79.53%)	690 (55.02%)	
Dyslipidemia				0.001
Yes	1,153 (9.03%)	1,008 (8.75%)	145 (11.56%)	
No	11,616 (90.97%)	10,507 (91.25%)	1,109 (88.44%)	

### 3.5 Nonlinear association between BMI and KOA in Chinese adults aged ≥45 years

**Figure 4:**
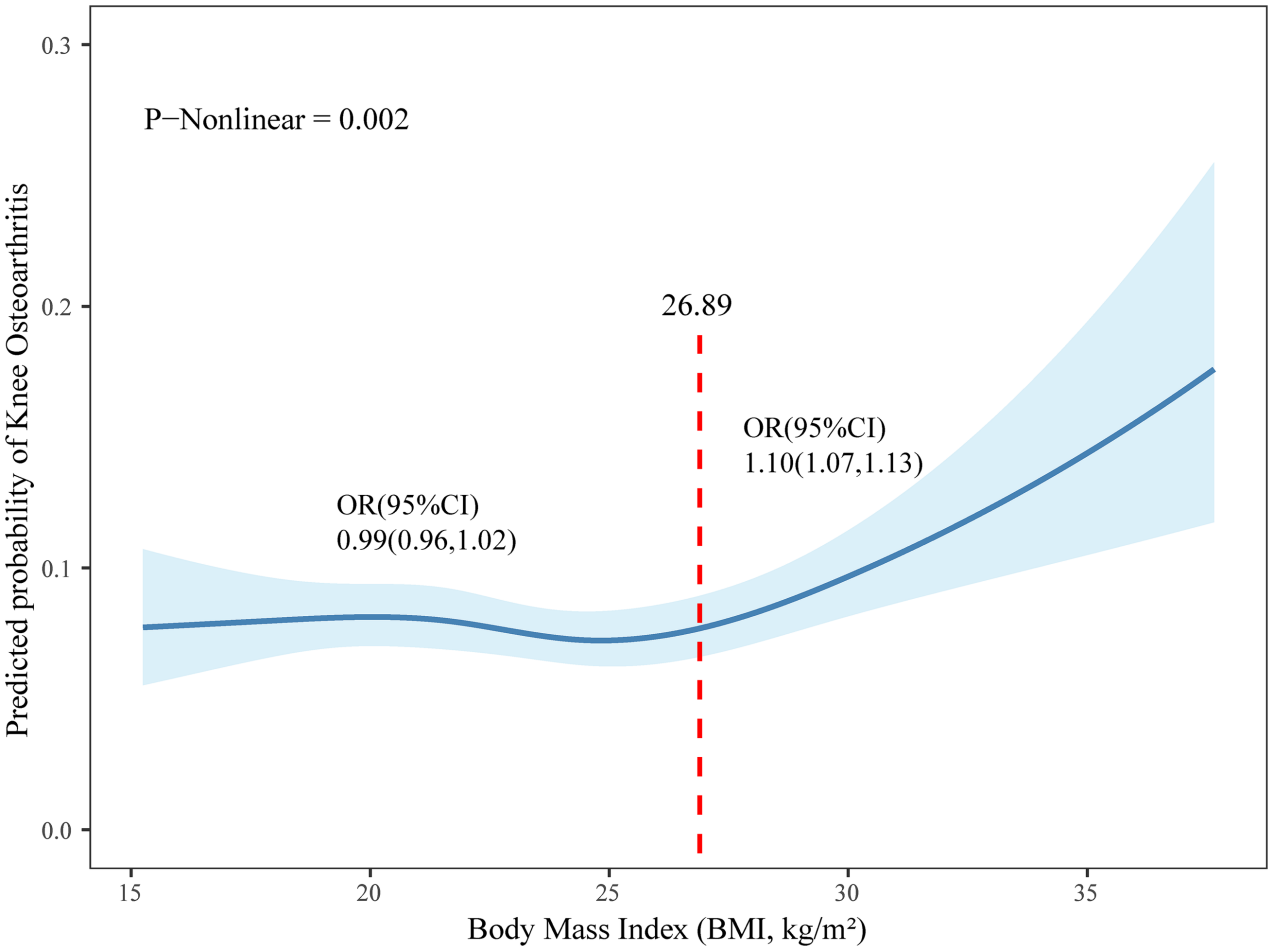
Nonlinear relationship between KOA and BMI in Chinese adults aged 45 and older.

Figure 4, based on CHARLS data, illustrated the correlation between BMI and the prevalence of KOA. After adjusting for age, sex, marital status, education, smoking, alcohol consumption, hypertension, diabetes mellitus, renal disease, digestive disorders, and dyslipidaemia, restricted cubic spline analysis revealed a consistent increase in KOA prevalence at higher BMI levels. Subsequent segmented regression analysis revealed a nonlinear relationship, with an inflection point at 26.89 kg/m^2^. In Model II, KOA prevalence remained relatively constant for BMI values below this threshold [odds ratio (OR) = 0.99, 95% confidence interval (CI): 0.96–1.02]. However, for each unit increase in BMI above this threshold, there was a corresponding 10% rise in KOA prevalence (OR = 1.10, 95% CI: 1.07–1.13, *p* < 0.001). The likelihood-ratio test showed that the piecewise specification significantly improved the model fit compared to the linear model (*p* = 0.005). These results emphasized the intricate, nonlinear association between BMI and KOA, highlighting the substantial increase in KOA risk associated with BMI values exceeding 26.89 kg/m^2^ (see Table A2).

### 3.6 Sensitivity analysis of the CHARLS

To assess the robustness of our results, we conducted a stratified analysis within the CHARLS cohort, employing a BMI threshold of 26.89 kg/m^2^ for stratification. Among participants with a BMI below 26.89 kg/m^2^, each additional 1 kg/m^2^ was not significantly associated with a change in KOA risk (OR = 0.97, 95% CI: 0.95–1.00). By contrast, for participants with a BMI of 26.89 kg/m^2^ or greater, each additional unit of BMI corresponded to a 10% increase in the risk of KOA (OR = 1.10, 95% CI: 1.03–1.16) (see Table A3).

## 4 Discussion

From 1990 to 2021, the burden of KOA in China has steadily increased, evolving into a major and increasingly complex public health challenge. Among modifiable risk factors, elevated BMI is of particular concern, as it represents a key driver of the KOA burden. High BMI increases mechanical stress on weight-bearing joints and promotes systemic inflammation, both of which accelerate disease progression (24). Given the substantial rise in obesity prevalence in China, future projections of the KOA burden are likely to be significantly underestimated unless this risk factor is explicitly considered (25). Furthermore, public awareness of early prevention and treatment remains persistently low, with many individuals only seeking medical attention when symptoms become apparent, thereby intensifying the long-term societal and clinical burden. Consequently, a rigorous appraisal of contemporary KOA epidemiology, coupled with the formulation of evidence-based weight management strategies, is imperative for preventing disease onset and attenuating progression.

This study provides an updated overview of the burden of KOA in China. In 2021, the ASIR, ASPR and ASDR were 406.42, 5,016.52 and 162.44 per 100,000 population, respectively. In 2021, 0.31% of total KOA DALYs were attributable to high BMI exposure, which is a 78.50% increase since 1990. DALYs attributed to BMI reached 1.11 million, marking a 369.28% increase since 1990. After age standardization, the ASDR increased by 91.68%. These findings are consistent with previous reports (7, 26) and confirm that the burden of KOA related to BMI has increased continuously between 1990 and 2021. Rapid economic development, coupled with shifts in dietary habits and reductions in physical activity, has driven population-level increases in BMI (27). As population ageing intensifies and the chronic disease burden escalates in China, KOA, a prototypical age-related disorder, has an incidence that rises steeply with age. This directly translates into a significant disease burden within this demographic (28). Stratified analysis across age groups revealed that the crude DALYs rate attributable to high BMI increased in every stratum between 1990 and 2021, albeit with fluctuations. Notably, the acceleration was most pronounced among adults aged 45 years and older, with a progressively steeper gradient at older ages, corroborating the observations of Li and Zhou (26). This is likely due to accelerated chondrocyte mitochondrial dysfunction with ageing, leading to metabolic imbalance within the joint matrix. Concurrently, the increased prevalence of metabolic syndrome among middle-aged and older populations amplifies the effects of joint damage through chronic inflammatory pathways. These epidemiological transitions highlight the need to prioritise adults aged ≥45 years in prevention and control programmes, requiring the development of robust and effective public health strategies to address this evolving challenge.

Furthermore, women consistently exhibited a higher burden of KOA and a steeper temporal increase in incidence, prevalence, and DALYs than men. This sex disparity is multifactorial. Anatomically, the female pelvis is wider and the Q-angle is larger, which amplifies mechanical loading across the tibiofemoral compartment and increases the risk of cartilage damage (29). Endocrine factors further magnify the risk. Dramatic hormonal fluctuations during the perimenopausal period, especially the abrupt decline in oestrogen after menopause, attenuate the chondroprotective effects of oestrogen and disrupt cartilage matrix turnover, accelerating joint degeneration and increasing susceptibility to KOA (30). Cyclical variations in sex steroid concentrations during the menstrual cycle and pregnancy also modulate ligamentous laxity and joint biomechanics, potentially compromising joint stability (31). Finally, age-related declines in bone mineral density and cartilage thickness are more pronounced in women, and the smaller, more fragile bony architecture of the female skeleton makes it more vulnerable to degenerative changes in weight-bearing joints, such as the knee (32). Collectively, these biological and biomechanical factors help explain the greater susceptibility of women to KOA.

Using CHARLS data, this study included 12,769 participants aged 45 years or older, among whom 1,254 (9.82%) were diagnosed with KOA—a prevalence consistent with previous estimates (33). We observed that KOA patients tended to have lower educational attainment, potentially because individuals with lower education levels are more likely to be employed in physically demanding occupations. Higher mechanical loads in such roles may increase susceptibility to joint cartilage damage, thereby elevating disease risk. A notably lower proportion of KOA sufferers were married, potentially because unmarried, divorced, or widowed individuals lack spousal companionship and care, leading to greater adverse health impacts. Smoking and alcohol consumption rates among KOA patients were comparatively low, possibly because patients voluntarily quit smoking and drinking after diagnosis—either following medical advice or due to self-perceived health risks—creating an illusion of “low exposure” in current surveys. Compared with individuals without KOA, those with KOA exhibited a significantly higher prevalence of hypertension, diabetes mellitus, renal disease, gastrointestinal disorders and dyslipidaemia. Both hypertension and KOA may share chronic systemic inflammation as a common risk factor, which could explain the elevated prevalence of hypertension in individuals with KOA (34). Hyperglycaemia accelerates joint inflammation and cartilage degradation via oxidative stress, activation of pro-inflammatory mediators and the accumulation of advanced glycation end-products (35). The chronic, low-grade inflammatory state that is characteristic of KOA, together with long-term use of non-steroidal anti-inflammatory drugs (NSAIDs), increases the incidence of renal and gastrointestinal disorders (36). With respect to lipid metabolism, high-density lipoprotein has been shown to suppress nuclear factor-κB activation and downregulate the expression of pro-inflammatory cytokines, such as interleukin-6 and tumour necrosis factor-*α*. This has the potential to attenuate synovial inflammation and cartilage catabolism. In contrast, low-density lipoprotein may accumulate in synovial fluid and cartilage, amplifying oxidative stress and inflammatory signalling and ultimately promoting cartilage breakdown and exacerbating joint symptoms (37). These findings highlight the importance of integrated, multimorbidity-oriented management of KOA patients to optimise therapeutic efficacy and improve long-term prognosis.

Consistent with prior studies (38, 39), overweight and obesity were significantly more prevalent among individuals with KOA. Meanwhile, the correlation analysis between KOA and BMI revealed that KOA prevalence increased with rising BMI, a trend that persisted even after adjusting for demographic and morbidity-related covariates. Piecewise regression modelling revealed a non-linear relationship between BMI and KOA, with a statistically derived inflection point at 26.89 kg/m^2^. Below this threshold, the prevalence of KOA remained essentially constant, whereas above it, each additional unit of BMI was associated with a 10% increase in the likelihood of developing the disease. Large-scale genetic correlation analyses corroborate this quantitative association: a one-standard-deviation increase in BMI was found to confer an odds ratio of 1.10 for KOA (40). Similarly, Lv et al. (41) observed a BMI odds ratio of 1.02 per unit, while a pooled meta-analysis estimated an excess risk of 35% for every 5-unit increment in BMI (42). Taken together, these findings confirm that a high BMI increases the risk of KOA. Excess weight increases mechanical loading across the knee joint, accelerating cartilage wear and degradation (43). Meanwhile, the strength of key muscle groups such as the quadriceps femoris decreases, leading to impaired joint alignment and stability (44). In addition to local mechanical stress, adipose tissue secretes pro-inflammatory cytokines, including interleukin-6, tumour necrosis factor-*α* and leptin, that upregulate matrix metalloproteinase activity, thereby exacerbating cartilage breakdown and synovial inflammation (45). In addition, an imbalance between leptin and adiponectin in obesity fosters a catabolic environment that favours cartilage destruction over repair and disrupts subchondral bone remodelling, thereby facilitating the initiation and progression of KOA (46). Precision public health initiatives should therefore implement personalised weight management protocols based on current BMI values to effectively reduce the population-level disease burden.

The World Health Organization advocates three complementary obesity management strategies: weight reduction, weight maintenance, and prevention of further weight gain, all aimed at sustaining a healthy body weight (13). There is robust evidence that structured weight management interventions for overweight or obese individuals can significantly reduce the incidence of KOA, highlighting their pivotal role in the primary prevention of this disease and reducing its impact on the population (47). In regions with a high concentration of individuals with an elevated BMI, comprehensive, multicomponent interventions are recommended. These should promote healthy diets with calorie restriction and portion control. It is also important to ensure adequate protein and micronutrient intake to support muscle and bone health. People should be encouraged to participate in joint-friendly activities such as swimming and cycling to support joint health and alleviate KOA-related pain. Early intervention is especially important for high-risk groups. Those with a BMI of 26.89 kg/m^2^ or higher should receive targeted, intensive management. This approach can lower their risk and help reduce the burden of KOA.

This study integrates GBD and CHARLS data to examine the relationship between BMI and KOA in Chinese adults aged 45 years and older; however, several limitations merit consideration. Firstly, the diversity of data sources may lead to variations in KOA diagnostic criteria and data collection methods, which could compromise the reliability of the findings. Secondly, the definition of KOA in the CHARLS cohort relies on self-reported data, which may introduce information bias due to inaccurate recall or socially desirable responses. Furthermore, the cross-sectional design of this study restricts our ability to infer a causal relationship between BMI and KOA risk. Even after adjusting for multiple covariates, unmeasured confounding factors could still influence the interpretation of the results. Meanwhile, given the limitations of cross-sectional design, we cannot rule out the reverse causality where KOA-induced pain and reduced activity may lead to increased BMI. Fourthly, BMI does not differentiate between fat and lean mass, nor does it capture variations in fat distribution. Therefore, using it as the sole indicator of body composition may not fully characterise an individual’s weight status or its influence on KOA risk. Finally, as this study is based on cross-sectional data from 2011, its findings may not accurately reflect the current relationship between BMI and KOA among middle-aged and older populations in China. These limitations suggest caution when extrapolating study findings and indicate directions for future research. For instance, longitudinal study designs could be employed, with repeated measurements of BMI and KOA incidence to clarify causal directionality, while utilising more comprehensive body composition and health indicators to provide a more detailed assessment.

## 5 Conclusion

BMI has emerged as a key modifiable risk factor for KOA-related DALYs. Convergent analyses of GBD 2021 and CHARLS data demonstrate a persistent and accelerating increase in the KOA burden attributable to high BMI across China, with a notable rise among adults aged ≥45 years. Specifically, a BMI threshold of ≥26.89 kg/m^2^ marks the point at which KOA risk escalates sharply in this population. This threshold should therefore be central to a multidimensional and integrated prevention framework that prioritises weight management, early intervention, and community-based health education. In addition, coordinated management of coexisting chronic conditions and the establishment of a precision prevention strategy stratified by age and sex are essential to curbing the continued expansion of the KOA burden in China.

## Data Availability

The original contributions presented in the study are included in the article/Supplementary material, further inquiries can be directed to the corresponding author.

## Footnotes

1
http://ghdx.healthdata.org/gbd-results-tool

## Glossary

OA: Osteoarthritis
KOA: Knee osteoarthritis
BMI: Body-mass index
OR: Odds ratio
GBD: Global Burden of Disease
CHARLS: China Health and Retirement Longitudinal Study
YLLs: Years of life lost
YLDs: Years lived with disability
DALYs: Disability-adjusted life years
TMREL: Theoretical minimum risk exposure level
PAF: Population attributable fraction
JRP: Joinpoint Regression Program
AAPC: Average annual percent change
ASIR: Age-standardized incidence rate
ASPR: Age-standardized prevalence rate
ASDR: Age-standardized DALYs rate
RCS: Restricted cubic splines
UI: Uncertainty interval
CI: Confidence interval
NSAIDs: Non-steroidal anti-inflammatory drugs
