# Supplementary material for: Association between BMI and knee osteoarthritis in Chinese adults aged 45 years and older: evidence from the 2021 Global Burden of Disease analysis and the China Health and Retirement Longitudinal Study
Source: Front Public Health. 2026 Jan 16;13:1738564. doi: 10.3389/fpubh.2025.1738564 (PMC12855550; doi:10.3389/fpubh.2025.1738564)
Supplement: Supplementary file 1 [file Table_1.docx]

Additional file


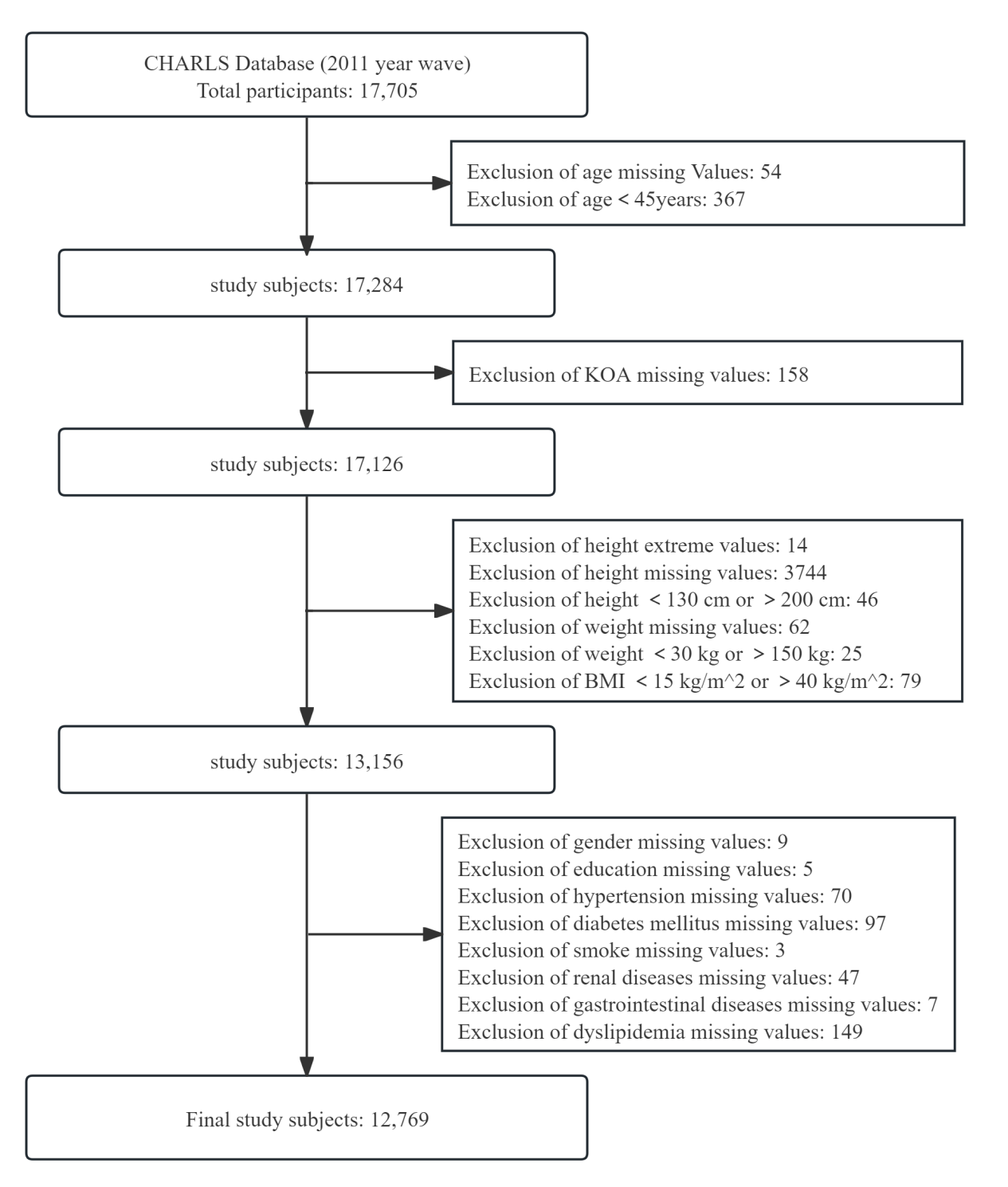


Figure A1: Inclusion and exclusion criteria for the CHARLS analytic cohort


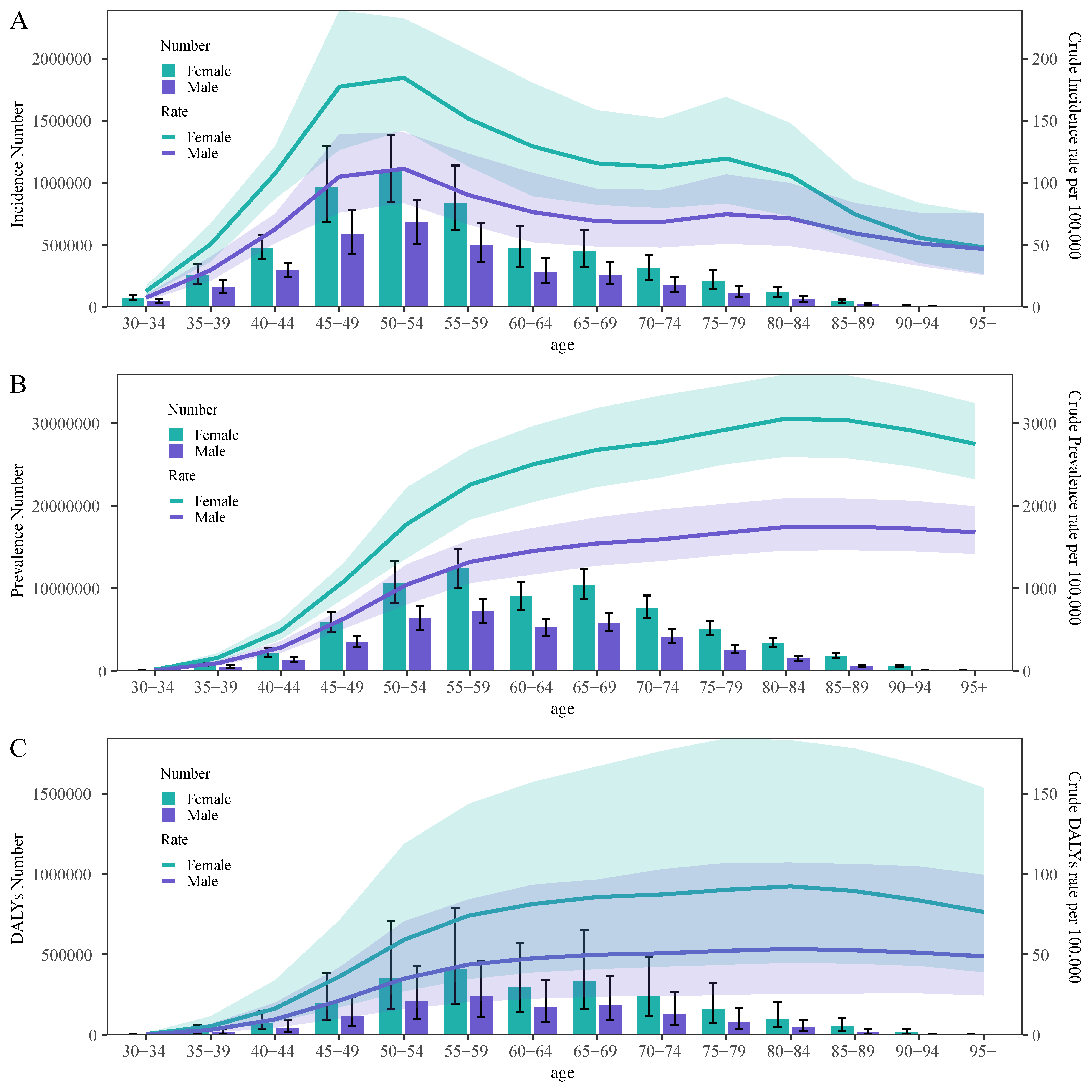


Figure A2: Sex- and age-stratified distribution of KOA incidence cases, prevalent cases, DALYs counts, and associated crude rates in China, 2021

| **Table A1**: Changes in the disease burden of KOA in China in 1990 and 2021 | | | | | | | |
| --- | --- | --- | --- | --- | --- | --- | --- |
|  | All ages cases  (n，95%UI) | | |  | Age standardized rates per 100,000 people  (n，95%UI) | | |
|  | Incidence | Prevalence | DALYs |  | Incidence | Prevalence | DALYs |
| 1990 | 3,650,857  (3,122,264,  4,200,440) | 41,044,009  (34,636,657,  47,406,915) | 3,650,857  (3,122,264,  4,200,440) |  | 377.93  (324.79,  434.28) | 4,667.29  (3,996.06,  5,359.85) | 151.24  (72.96,  291.47) |
| 2021 | 8,512,397  (7,279,974,  9,840,885) | 109,575,472  (92,723,351,  126,639,049) | 8,512,397  (7,279,974,  9,840,885) |  | 406.42  (348.70,  467.23) | 5,016.52  (4,256.22,  5,758.38) | 162.44  (78.35,  314.13) |
| Relative change (%) | 1.33 | 1.67 | 1.65 |  | 0.08 | 0.07 | 0.07 |
| DALYs: disability-adjusted life years; 95%UI: 95% uncertainty interval | | | | | | | |

| Table A2: Segmented regression models in CHARLS. | | |
| --- | --- | --- |
| Model II |  | *P* value |
| Breakpoint (k) | 26.89 |  |
| Segment | OR (95%*CI*) |  |
| Effect 1: < k | 0.99 (0.96, 1.02) | 0.391 |
| Effect 2: > k | 1.10 (1.07, 1.13) | <0.001 |
| Log-likelihood ratio test |  | 0.005 |

| Table A3: Subgroup analysis of BMI and KOA risk according to the CHARLS | | | | | | | |
| --- | --- | --- | --- | --- | --- | --- | --- |
| Subgroup | BMI<26.89 | | |  | BMI≥26.89 | | |
|  | n | OR (95%*CI)* | *P* value |  | n | OR (95%*CI*) | *P* value |
| Overall | 10,612 | 0.97(0.95,1.00) | 0.044 |  | 2,157 | 1.10(1.03,1.16) | 0.002 |
| Age |  |  |  |  |  |  |  |
| T1 | 3,427 | 0.95(0.90,1.01) | 0.104 |  | 900 | 1.01(0.91,1.12) | 0.798 |
| T2 | 3,733 | 0.99(0.95,1.03) | 0.543 |  | 747 | 1.12(1.02,1.23) | 0.015 |
| T3 | 3,452 | 1.01(0.97,1.05) | 0.73 |  | 510 | 1.17(1.04,1.31) | 0.007 |
| Gender |  |  |  |  |  |  |  |
| Male | 5,309 | 0.97(0.93,1.01) | 0.176 |  | 792 | 1.16(1.02,1.30) | 0.014 |
| Female | 5,303 | 0.96(0.93,0.99) | 0.009 |  | 1,365 | 1.06(0.99,1.14) | 0.086 |
| Education |  |  |  |  |  |  |  |
| Illiterate | 3,010 | 1.00(0.96,1.05) | 0.849 |  | 556 | 1.09(0.98,1.21) | 0.093 |
| Primary school | 4,402 | 0.99(0.95,1.03) | 0.646 |  | 815 | 1.05(0.95,1.15) | 0.343 |
| Middle school | 2,089 | 0.97(0.90,1.04) | 0.403 |  | 517 | 1.16(1.02,1.31) | 0.021 |
| High school and above | 1,111 | 0.97(0.85,1.11) | 0.64 |  | 269 | 1.20(0.94,1.48) | 0.111 |
| Marital status |  |  |  |  |  |  |  |
| Married | 8,673 | 0.98(0.96,1.01) | 0.256 |  | 1,872 | 1.10(1.03,1.17) | 0.003 |
| Others | 1,939 | 0.95(0.90,1.01) | 0.091 |  | 285 | 1.07(0.89,1.27) | 0.43 |
| Smoke |  |  |  |  |  |  |  |
| Yes | 4,497 | 0.97(0.93,1.01) | 0.159 |  | 617 | 1.20(1.07,1.35) | 0.002 |
| No | 6,115 | 0.97(0.94,1.00) | 0.03 |  | 1,540 | 1.06(0.99,1.13) | 0.113 |
| Drink |  |  |  |  |  |  |  |
| Yes | 2,787 | 0.98(0.92,1.04) | 0.46 |  | 401 | 1.11(0.94,1.29) | 0.182 |
| No | 7,825 | 0.97(0.94,1.00) | 0.042 |  | 1,756 | 1.09(1.02,1.16) | 0.007 |
| Hypertension |  |  |  |  |  |  |  |
| Yes | 2,126 | 0.93(0.88,0.97) | 0.003 |  | 953 | 1.10(1.01,1.19) | 0.022 |
| No | 8,486 | 0.98(0.95,1.01) | 0.112 |  | 1,204 | 1.08(0.98,1.18) | 0.109 |
| Diabetes mellitus |  |  |  |  |  |  |  |
| Yes | 494 | 0.90(0.82,1.00) | 0.053 |  | 250 | 0.93(0.76,1.11) | 0.431 |
| No | 10,118 | 0.98(0.95,1.00) | 0.06 |  | 1,907 | 1.12(1.05,1.19) | <0.001 |
| Renal diseases |  |  |  |  |  |  |  |
| Yes | 681 | 0.99(0.93,1.06) | 0.83 |  | 131 | 1.05(0.88,1.23) | 0.583 |
| No | 9,931 | 0.97(0.95,1.00) | 0.066 |  | 2,026 | 1.10(1.03,1.17) | 0.003 |
| Gastrointestinal diseases |  |  |  |  |  |  |  |
| Yes | 2,518 | 1.00(0.96,1.04) | 0.862 |  | 403 | 1.17(1.05,1.32) | 0.007 |
| No | 8,094 | 0.98(0.94,1.01) | 0.181 |  | 1,754 | 1.10(1.02,1.18) | 0.011 |
| Dyslipidemia |  |  |  |  |  |  |  |
| Yes | 729 | 0.97(0.88,1.07) | 0.524 |  | 424 | 1.16(1.04,1.30) | 0.007 |
| No | 9,883 | 0.97(0.95,1.00) | 0.031 |  | 1,733 | 1.06(0.98,1.14) | 0.117 |
